# Genome wide identification and characterization of microsatellite markers in black pepper (*Piper nigrum*): A valuable resource for boosting genomics applications

**DOI:** 10.1371/journal.pone.0226002

**Published:** 2019-12-13

**Authors:** Ratna Kumari, Dhammaprakash Pandhari Wankhede, Akansha Bajpai, Avantika Maurya, Kartikay Prasad, Dikshant Gautam, Parimalan Rangan, M. Latha, Joseph John K., Suma A., Kangila V. Bhat, Ambika B. Gaikwad

**Affiliations:** ICAR-National Bureau of Plant Genetic Resources, New Delhi, India; Beijing Forestry University, CHINA

## Abstract

Black pepper is one of the most valued and widely used spices in the world and dominates multi-billion dollar global spices trade. India is amongst the major producers, consumers and exporters of black pepper. In spite of its commercial and cultural importance, black pepper has received meagre attention in terms of generation of genomic resources. Availability of markers distributed throughout the genome would facilitate and accelerate genetic studies, QTL identification, genetic enhancement and crop improvement in black pepper. In this perspective, the sequence information from the recently sequenced black pepper (*Piper nigrum*) genome has been used for identification and characterisation of Simple Sequence Repeats (SSRs). Total 69,126 SSRs were identified from assembled genomic sequence of *P*. *nigrum*. The SSR frequency was 158 per MB making it, one SSR for every 6.3 kb in the assembled genome. Among the different types of microsatellite repeat motifs, dinucleotides were the most abundant (48.6%), followed by trinucleotide (23.7%) and compound repeats (20.62%). A set of 85 SSRs were used for validation, of which 74 produced amplification products of expected size. Genetic diversity of 30 black pepper accessions using 50 SSRs revealed four distinct clusters. Further, the cross species transferability of the SSRs was checked in nine other *Piper* species. Out of 50 SSRs used, 19 and 31 SSRs were amplified in nine and seven species, respectively. Thus the identified SSRs may have application in other species of the genus *Piper* where genome sequence is not available yet. Present study reports the first NGS based genomic SSRs in black pepper and thus constitute a valuable resource for a whole fleet of applications in genetics and plant breeding studies such as genetic map construction, QTL identification, map-based gene cloning, marker-assisted selection and evolutionary studies in *Piper nigrum* and related species.

## Introduction

Spices have been an important ingredient of food for human consumption all over the world since time immemorial. Among the spices, black pepper (*Piper nigrum* L.), is the most widely used spice in the world and therefore commercially the most important one, no wonder it is known as ‘the king’ of spices [1].

Black pepper (2n = 4x = 52) is a perennial woody climbing vine of the Piperaceae family. The berries (dried mature fruits) are of economic importance owing to its pungency and flavour, attributed to alkaloid Piperine and volatile oil, respectively [1,2]. Black pepper is used in human diet as spice and seasoning as well as for several other purposes such as traditional medicines, preservatives and perfumery [3]. Piperine, an alkaloid from black pepper is reported to possess cytotoxic activity towards tumour cell lines [4], antipyretic, analgesic, anti-inflammatory activities and is also shown to protect against chemical carcinogens [5]. Piperine in diet, however, is known to stimulate digestive enzymes and thus enhance digestion [6] and therefore black pepper has been an important ingredient of food preparations in different parts of the world.

In commercial perspective, the global market of spices is estimated to be USD 12 billion, of which black pepper constitutes a major share with India among the leading exporters [7]. However, there is still tremendous potential and scope to increase the larger share of India in global market.

Black pepper has its origin in Western Ghats of India (south western regions of India) from where it spread to Indonesia, Malaysia and other South-East Asian countries [8]. Western Ghats, especially the Kerala state of India harbours the maximum genetic diversity of black pepper [8]. However, there have been a very few studies on genetic diversity using molecular markers [9–13] primarily due to limitations in genetic resources availability. Therefore black pepper remained largely untouched from genomic interventions.

Advances in plant genomics have facilitated deeper insights to crop diversity at species as well as gene levels [14]. Availability of genomic resources in the form of DNA based markers, is expected to accelerate basic research such as genetic map construction, QTL/gene mapping, comparative genomics and ultimately molecular breeding which expedite pace of varietal development [15–19]. Among the DNA based markers, micro-satellite or Simple Sequence Repeat markers are a preferred marker system of researchers owing to their advantages such as reproducibility, multi-allelic and co-dominant nature and genome coverage [20]. Additionally, SSRs are also amenable to high throughput genotyping platforms, albeit with the lower automation efficiency than SNP genotyping technologies. Microsatellites or SSRs are tandem repeats of 1 to 6 nucleotide found interspersed in the genome (both coding and non-coding regions) [21].

In black pepper there have been only a few reports of generation of SSRs [13, 22]. Lately, transcriptome based approaches have been employed for generation of SSRs in black pepper [23–24]. These approaches largely represent only expressed portion of the genes and are restricted to only the genic regions of the genome thus limiting their applications in linkage map construction, diversity and evolutionary studies.

The advances in next generation sequencing (NGS) technologies have accelerated marker generation with higher efficiency [25–26]. This also has expedited identification of simple sequence repeats (SSR) and their flanking regions for generation of PCR based markers. NGS has been used in recent years for generation of genomic SSRs in wide range of plants species such as watermelon [27], cotton [28], finger millets [29], Foxtail Millet [30], faba bean [26], progenitors of peanut, *Arachis duranensis* and *A*. *ipaensis* [31] and Maqui [32]. The flanking regions of SSRs are usually conserved across related species or genera and hence the primers developed in one species can be tested for amplification across related species and genera, and is known as cross species amplification or transferability [33]. This saves time, effort and resources in the development of SSR markers in related species. In the recent past, cross species amplification has been used in several crops for genetic and evolutionary studies [34–40].

We recently have sequenced the draft genome of the black pepper (*P*. *nigrum*) using the Illumina, PacBio (NCBI GenBank: PRJNA412127) and IRYS sequencing platforms to generate a draft genome comprising of 916 scaffolds at a genome coverage of 80X (manuscript under preparation) and used the sequence information for genome wide mining and characterization of SSR in black pepper. This is the first report of large scale generation of genomic SSR sequences in black pepper. The SSR markers developed from *Piper nigrum* were tested for cross amplification in nine *Piper* species. The polymorphic SSR markers identified in the present study can be directly used in other species for diversity analysis and genetic and evolutionary studies especially in the species where they are not available.

## Materials and methods

The plant material included 30 accessions of *Piper nigrum* and eighteen accessions belonging to ten *Piper* species (including two accessions of *P*. *nigrum*). The leaf samples were collected from ICAR-NBPGR regional station, Thrissur, Kerala, India. The leaf samples were fixed in liquid nitrogen and stored at -80°C until extraction. The list of accessions (*P*. *nigrum* and *Piper* sps.) used for diversity analyses and cross species transferability study have been shown in Table 1 and Table 2, respectively.

**Table 1:** List of *Piper nigrum* accessions used for validation of SSRs and diversity analysis.

S. No.	Accession no.	Cultivar name	Village	District
1	IC85318	Nadan	Kuttampuzha, Adimali	Idukki
2	IC85320	Karimunda	Puyamkutti	Idukki
3	IC85354	Vattamundi	Nedunkandam	Idukki
4	IC85375	Narayakkodi	55mile Peermed	Idukki
5	IC85386	Malamundi	Thadiyanpad	Idukki
6	IC85387	Karimunda	Thadiyanpad	Idukki
7	IC85388	Neelamundi	Thadiyanpad	Idukki
8	IC85396	Chomala	Mekkazhoor	Pathanamthitta
9	IC85397	Karimunda	Perinadu	Pathanamthitta
10	IC85402	Palikkodi	Kochandi	Pathanamthitta
11	IC85410	Thottamunda	Chittar	Pathanamthitta
12	IC85418	Karivalli	Konni	Pathanamthitta
13	IC85433	Cholakkodi	Chambakkara	Kottayam
14	IC85434	Ottanadan	Kallara	Kottayam
15	IC85543	Kureidmundi	Panniyur	Cannanore
16	IC360238	Valiyaramunda	Arikkakavu	Idukki
17	IC360239	Narayakkodi	Padayinippara	Idukki
18	IC266410	Karimunda	Mandiram, Ranni	Pathanamthitta
19	IC266409	Kottakkodi	Mandiram, Ranni	Pathanamthitta
20	IC266446	Vally	Panniyur, KAU	Cannanore
21	IC266457	Perumkkodi	Panniyur, KAU	Cannanore
22	IC373832	Thottumuriyan	Mavila	Kollam
23	IC373837	Annarvarayan	Ummannoor, Kottarakar	Kollam
24	IC373831	Narayakkodi	Ariyankavu, Thenmala	Kollam
25	IC373755	Vadakkan	Kulathur, Kottarakara	Kollam
26	IC373782	Munda	Veerapuli	Kanyakumari
27	TCR 353			
28	P1	Panniyur 1		
29	TCR 229			
30	TCR 383	Karimunda		

**Table 2:** List of accessions in different *Piper* species used for cross species amplification.

S. No.	Species Name	TCR No.
1	*Piper nigrum*	TCR 419
2	*Piper nigrum*	TCR 8
3	*Piper longum*	TCR 212
4	*Piper longum*	P25
5	*Piper arboreum*	TCR 267
6	*Piper arboreum*	*Piper arboreum*
7	*Piper argyrophyllum*	TCR 302
8	*Piper argyrophyllum*	TCR 365
9	*Piper attenuatum*	TCR 171
10	*Piper betel*	TCR 166
11	*Piper betel*	TCR357
12	*Piper betel*	*Piper betel* Lakshdweep
13	*Piper chaba*	TCR 149
14	*Piper chaba*	TCR265
15	*Piper hymenophyllum*	TCR345
16	*Piper trichostachyon*	TCR363
17	*Piper trichostachyon*	TCR279
18	*Piper wallichi*	*Piper wallichi* Andaman

### Identification of microsatellites from *Piper nigrum*

Total genomic DNA was isolated from the leaf samples using CTAB extraction method [41]. The purified DNA was checked on 1% agarose gel and quality checked on Nanodrop (DS-11 spectrophotometer, DeNovix, Wilmington, Delaware). Finally DNA was quantified using Qubit 2.0 fluorescence spectrophotometer (Life Technologies) for preparing genomic libraries. Draft genome sequence of black pepper (unpublished data) generated using short reads, long reads and optical mapping, assembled into less than 1200 scaffolds with a N50 of more than 5 Mb was used for mining microsatellites.

The genome sequence of *Piper nigrum* was searched for presence of different microsatellite repeats from di to hexa nucleotide simple as well as complex repeats following the default parameter of MISA -MIcroSAtellite identification tool (http://pgrc.ipk-gatersleben.de/misa/). The SSRs were identified from the draft genome using MISA perl scripts [42]. The search criteria included minimum of six repeats of dinucleotides, minimum five repeats for trinucleotides, tetranucleotides, pentanucleotides and hexanucleotides. The identified SSRs were then classified into perfect and compound and on the basis of type of repeat motif present. The genome sequence annotation (.GFF files) was used for defining SSRs in the genic and intergenic regions.

### SSR primer design and validation

The primers were designed from the flanking sequences of identified SSRs using software Primer 3 [43]. Primers were designed for 66997 of the 69126 identified SSRs. 85 SSR primer pairs were synthesized for wet lab validation. The genomic DNA was isolated from leaf tissue using CTAB DNA extraction method. The quality of DNA was checked on 1% agarose gel and quantified using nanodrop spectrophotometer. The PCR reaction consisted of total volume of 20μl comprising of 1X PCR buffer, 2.5mM MgCl_2_, 1μM primer, 0.2mM of each dNTPs, 1U Taq DNA polymerase (NEB) and 15 ng template DNA. The PCR reaction was carried out in thermal cycler (Eppendorf) with the following program: Initial denaturation at 95°C for 5min followed by 35 cycles of denaturation at 95°C for 1min, annealing at 50–58°C for 1min and extension at 72°C for 1min followed by final extension at 72°C for 10min. The amplification products were resolved on 3% metaphor gel. A 50bp DNA ladder was used as size standard. For diversity analyses, amplified products were resolved on QIAxcel multi-capillary system using QIAxcel High Resolution Kit 1200 (QIAGEN, No 929002), 50-800bp v2.0 Qx DNA size marker (QIAGEN, No 929561) and 15bp/1000bp Qx alignment marker (QIAGEN, No. 929521). PCR products were separated with high resolution run method OM700 with a sample injection time of 10 seconds. The allelic sizes of each sample were resolved and calculated in the form of gel profiles and peaks using QIAxcel Screengel Software (QIAGEN, v1.5).

### Data analysis

The SSR amplification products (bands) were scored across the lanes according to their molecular weight. The alleles were scored as present (1) or absent (0) in the binary format to assess diversity and genetic relationship among the *P*. *nigrum* accessions. The data was analysed using software program NTSYS-pc ver. 2.1 [44]. The Jaccard’s similarity index was calculated between pairs of genotypes. The genotype x allele similarity index was subjected to UPGMA (unweighted pair group method for arithmetic mean) analysis and a dendrogram was generated. To study cross species transferability of SSR, the bands were scored across the ten *Piper* species (including *P*. *nigrum*) and scored as present (+) and absent (–).

## Results

### Identification of microsatellites from *Piper nigrum*

**Fig 1:**
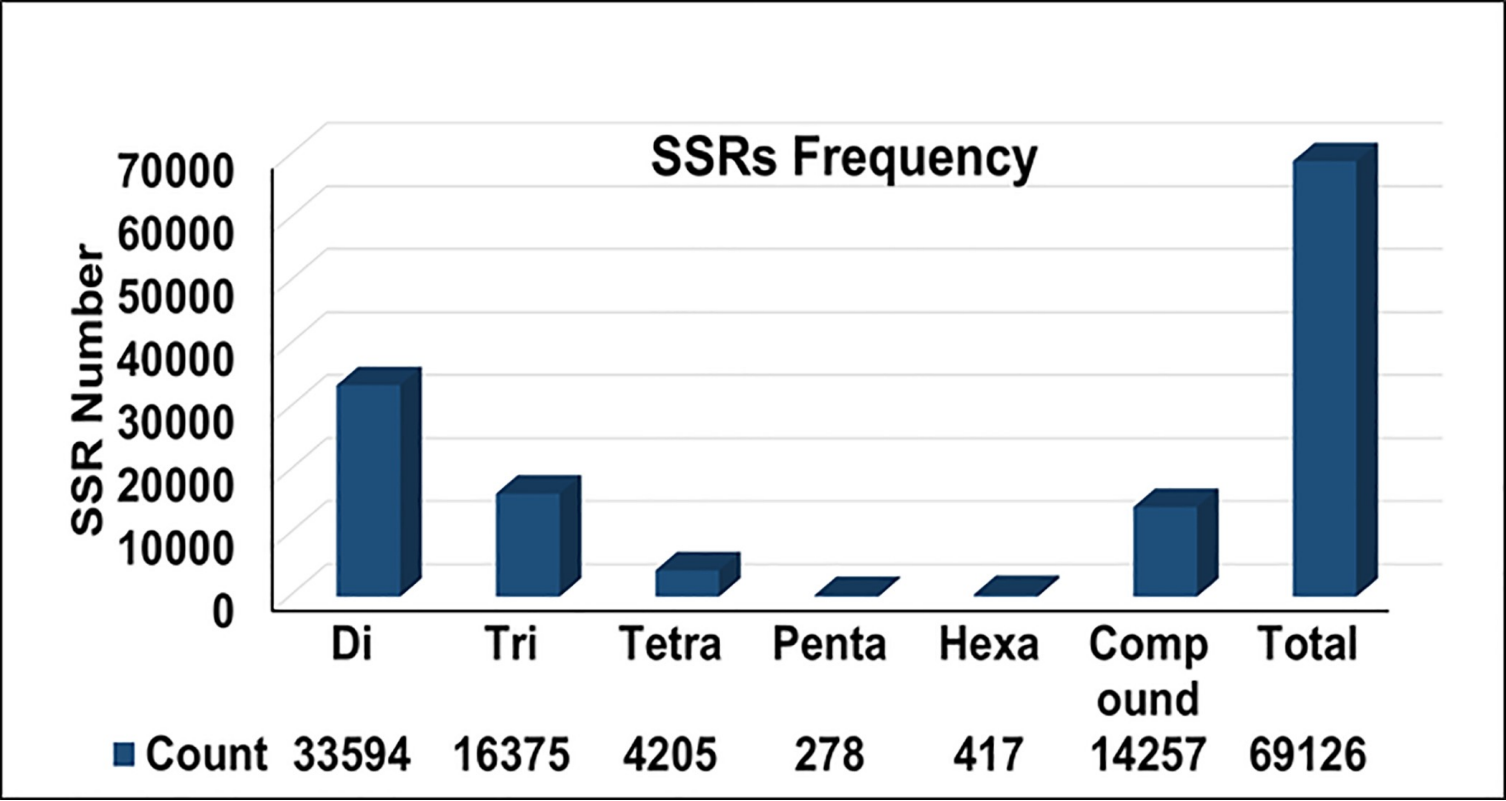
Distribution of simple sequence repeats in the draft genome sequence of black pepper (*Piper nigrum*).

**Fig 2:**
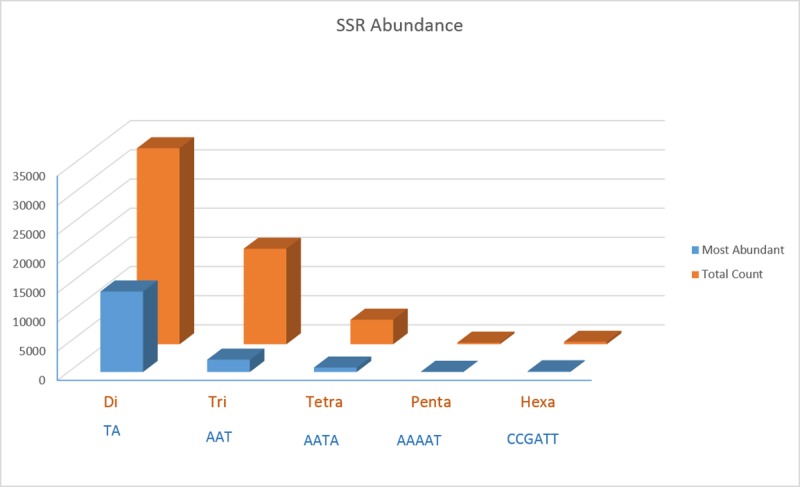
Abundance of specific SSR motifs in di- to hexanucleotides repeats in the *Piper nigrum* genome.

**Fig 3:**
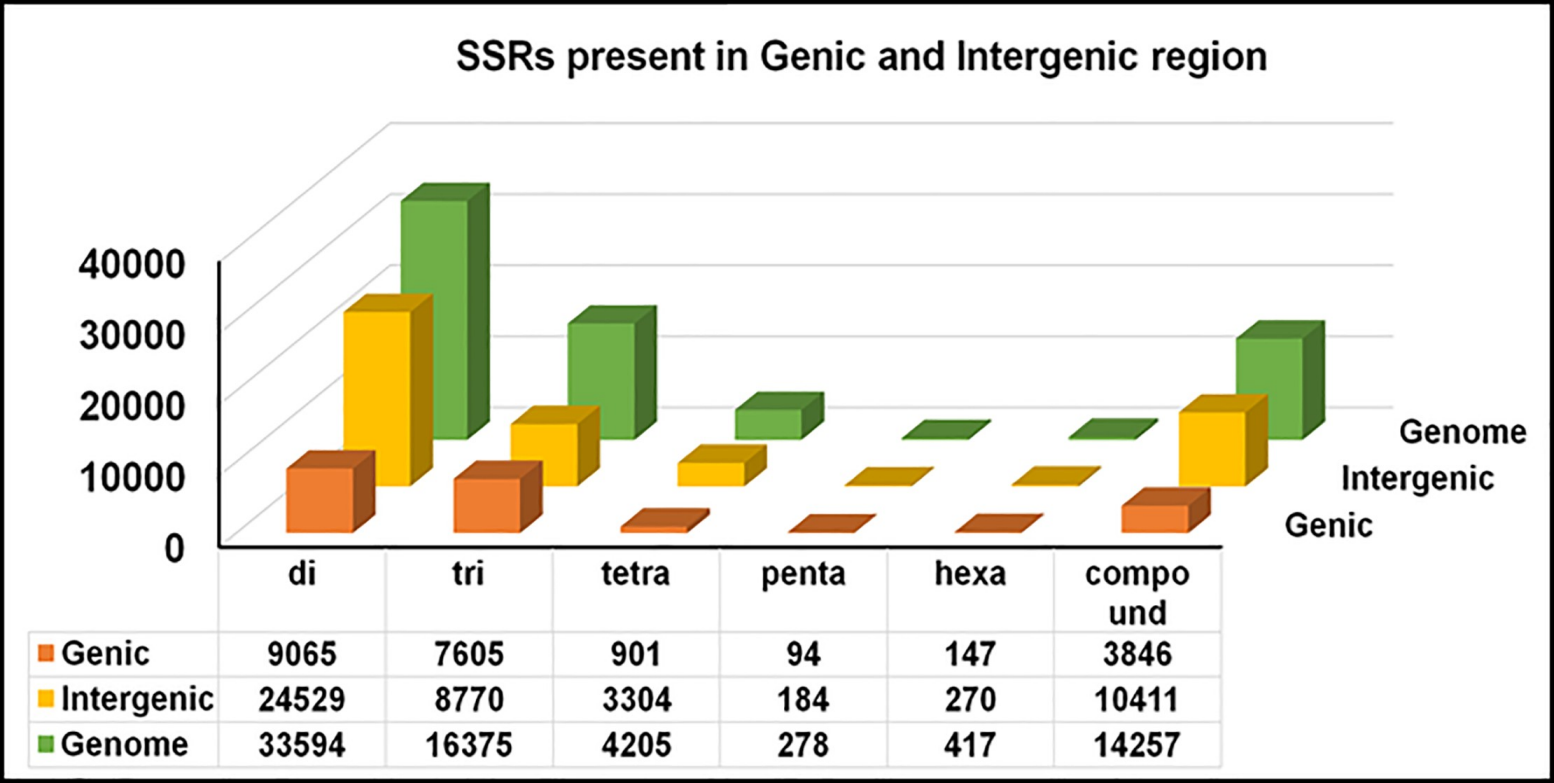
Distribution of SSRs identified in genic and intergenic regions of *Piper nigrum*.

The assembled genome sequence of *Piper nigrum* was searched for presence of different microsatellite repeats from dinucleotides to hexanucleotides, simple as well as complex repeats. Total 69,126 SSRs were identified from 430 Mb assembled genome sequence of *P*. *nigrum*. The frequencies of SSRs were 158 per Mbp making it one SSR for every 6.3 kb in the assembled genome sequence. From the total 69,126 SSRs, 54,869 (79.4%) were perfect SSRs and 14,257 (20.6%) were compound SSRs. Among the perfect SSRs, dinucleotide repeats were highest in number 33,594 (61.2%), followed by trinucleotide 16,375 (29.8%) and tetranucleotide repeats 4205 (7.6%). Pentanucleotide repeats were the least in number 278 (0.5%) (Fig 1). Among different types of repeats, it was observed that in each type, one particular motif was predominant. From the identified SSRs, 41% of the total dinucleotide repeats (33594) was ‘TA’, 12.9% of the trinucleotide repeats (16375) was ‘AAT’, 18.1% of the tetranucleotides repeats (4205) was ‘AATA’, 11.9% of the pentanucleotide repeats (278) was ‘AAAAT’ and 29.5% of the hexanucelotide repeats (417) was ‘CCGAAT’ (Fig 2). In case of compound SSRs, a majority (71.9%) were interrupted whereas 28.1% were uninterrupted compound repeats. In terms of genic and inter genic regions, distribution of SSRs were 21658 and 47468 respectively. Among the individual repeats type, distribution of dinucleotide, trinucleotide and tetra nucleotide SSRs in genic regions were 27%, 46.4% & 21.4%, respectively (Fig 3). The penta and hexa repeats were 33.8% and 35.2%, respectively, whereas the compound SSRs were present to the tune of 30% of the total in the genic regions.

**Fig 4:**
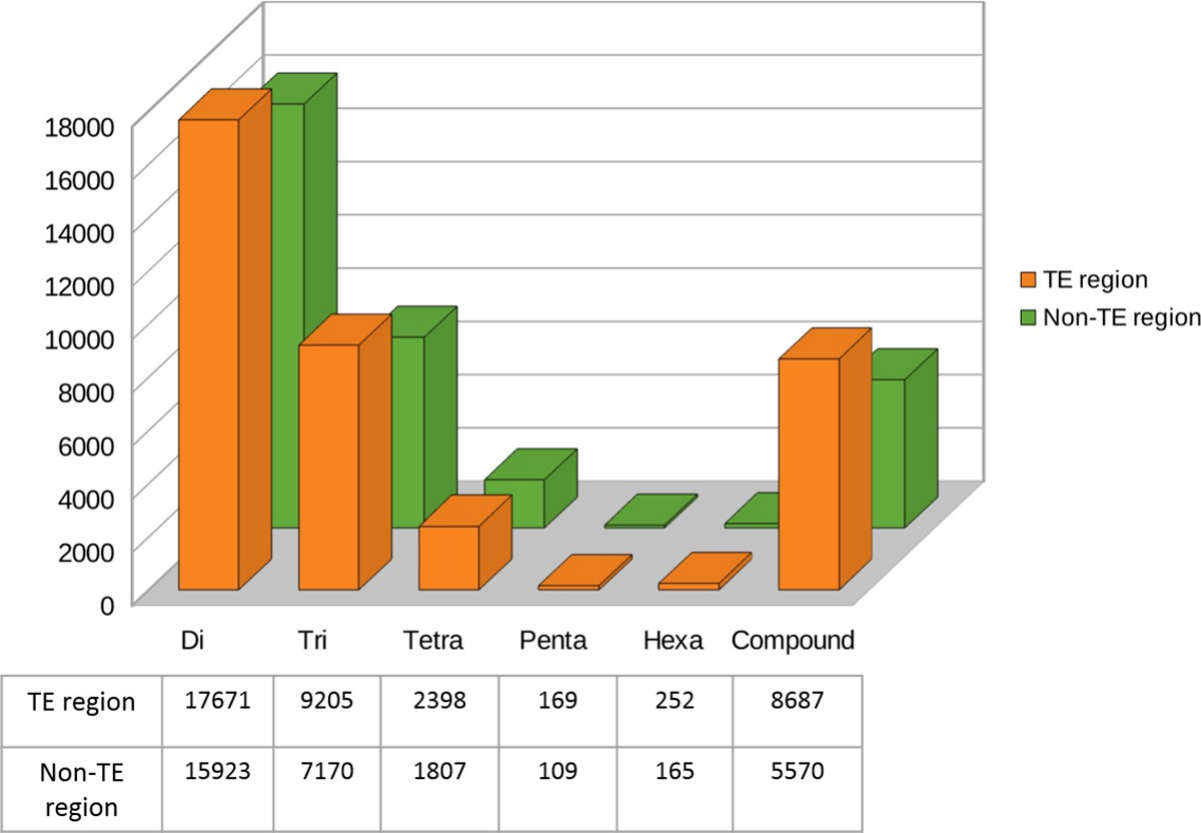
Distribution of SSRs identified in regions with transposable elements in *Piper nigrum*.

There was also significant presence of SSRs in transposable elements. From the total SSRs identified 56% (38382) were from the region with transposable elements. The proportionate abundance of each repeat type (di- to hexa-nucleotide and compound repeats) followed a similar pattern with more number of microsatellites being present in the transposable element region than in the non-transposable element region **(**Fig 4).

### Development and validation of SSR primers

**Fig 5:**
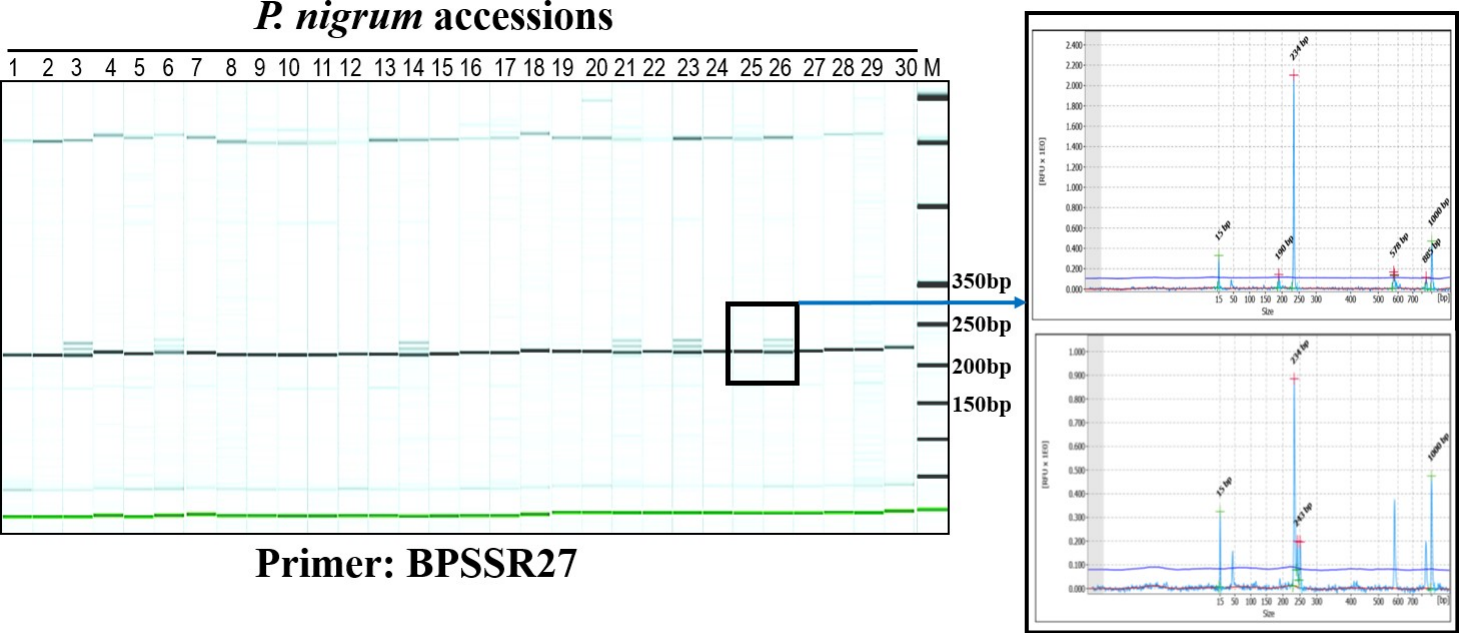
Validation of SSR markers in germplasm accessions of black pepper (*Piper nigrum*). Gel image of PCR amplification of SSR marker BPSSR27 on 30 germplasm accessions of *P*. *nigrum* as captured on QIAxcel ScreenGel software is shown on the left side. Numbers refer to accession numbers as mentioned in Table 1. The lane marked ‘M’ is DNA molecular weight standard 50-800bp v2.0 Qx DNA size marker. A representative electropherogram showing allele sizes of 234 bp (sample 25) and 234, 243 and 249bp (sample 26) has been shown on right side of the figure.

**Fig 6:**
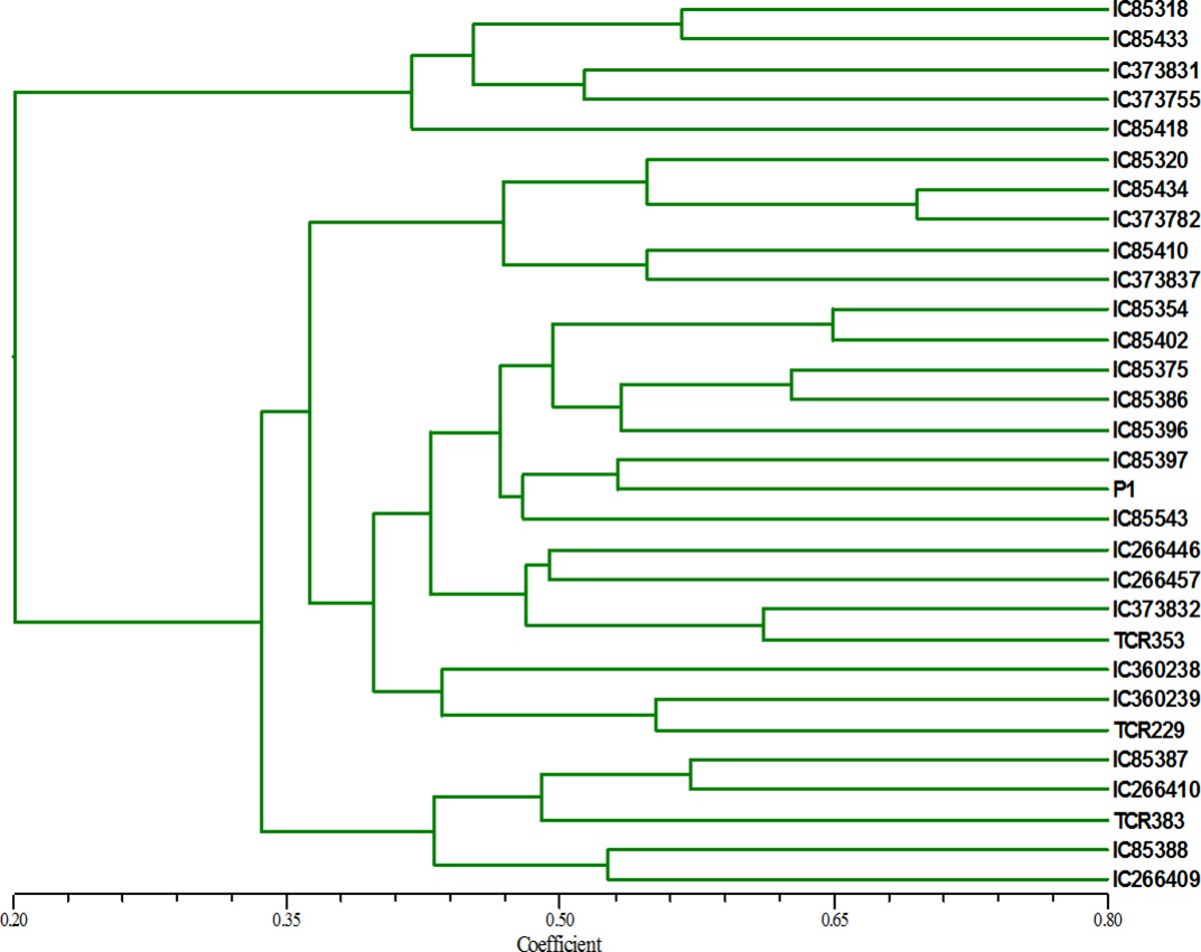
Genetic relationship of germplasm accessions as revealed by SSR markers in black pepper. Dendrogram was constructed using SSR profiles for 30 black pepper (*Piper nigrum*) accessions. The pairwise Jaccard’s similarity coefficients was used for construction of phylogenetic tree.

In order to use the genome wide SSRs mined from the black pepper genome as PCR based SSR markers, forward and reverse primers were designed for 66997 SSRs using Primer3 program. For each SSR, five different sets of primers were designed (S1 Table). In order to validate the identified SSRs, a set of 85 SSR primers were custom synthesized (Table 3) and checked for PCR amplification. Out of 85 primer pairs, 74 primer pairs produced amplification product of expected size. For diversity analysis, 50 of the validated 74 SSR markers were used on 30 landraces of black pepper. All these 50 SSR loci were polymorphic in nature. A representative amplification profile of 30 black pepper accessions with SSR primer BPSSR27 as resolved on QIAxcel multi-capillary system is shown in Fig 5. From the 50 primers, a total of 215 alleles were detected with an average of 4.3 alleles per locus. The allelic data was used to calculate pairwise Jaccard’s similarity coefficients that ranged from 0.08 to 0.69 with an average of 0.34. The similarity index was subjected to UPGMA analysis and a dendrogram was generated. The dendrogram grouped 30 landraces into four major clusters (Fig 6).

**Table 3:** List of 74 validated SSR primer pairs from black pepper used for diversity analysis and cross species transferability.

Primer ID	SSR motif	Forward primer (5’-3’)	Reverse primer (5’-3’)	Annealing Temperature (ºC)	Product size (bp)
BPssr_1	GT	GCTGGGTCACACATAGGTCC	TTGAGGCTATGGCGGTAAGT	57	277
BPssr_2	TC	TTAGCAAAGCGCAAACCCAC	ACCAACTGATCGTGACCGTC	57	272
BPssr_3	GA	TAGGCGGTGGCAAAACAGT	TGCATACCCACCACATACGT	57	280
BPssr_4	GA	CTTCTGTGATGGGCGAAGGT	GTGATGACCAGCTCTTGCCT	56	231
BPssr_5	AC	GGCCCCAACTCTCCTACAAC	CCAACACACACACATCAGCC	57	167
BPssr_6	TG	TTGTGCATGTGTGGAGGTGT	CGCCAGCGTTGTCCTACATA	57	214
BPssr_7	TA	GGGAGAGAAGGGGTGAGATG	CCCTCTCTTATCAATGCGCCT	57	158
BPssr_8	CA	CACTATTGTCGGGATGGCCA	ACCGATGACGTCCTCGACTA	56	153
BPssr_9	GA	TGTTCTAGAGCCTGGACCCA	TTCCTGTGCGTTGGTAGCAT	54	162
BPssr_10	TC	AGGCGGTAATGGATTGGGTG	GTTCTTCTCGCCTTGGTCCA	56	201
BPssr_11	TG	CCTACCGAGAGCTTGAGCAC	GCAGTCGGGCACTCTACATT	57	214
BPssr_12	AT	CCCAGGTTGAGGGTGGAAAA	AGTCGTAGCGGGAAAAACAGA	57	193
BPssr_13	AC	ACGGTGATGTCGGTTCCATT	TCCTCTTCGGCATGGTACCT	56	218
BPssr_14	GT	CACTGCTGCCCTAGTTCGAA	ATCACCATCCACTCGGTGTG	55	180
BPssr_15	AT	GTTGCACCGACCATGCTTTT	AGGAGCCGAGAAAGCAGAAG	55	176
BPssr_16	TA	GTTGAGCCCGTCACATACCA	GCTCCTTTCTGACCTGCCAT	55	216
BPssr_17	AT	CCATTCGCCGACCCATATGA	ATGATCAACCCGGCGAACTT	57	210
BPssr_18	AT	TGCCTATCGTTATTTTTGTGAGCT	AAGTTGGCTTCCCACGAGAG	54	220
BPssr_19	TC	ATGCCCGGTATGACTTGGAC	GACGTGGAATGCTGCCTAGA	55	192
BPssr_20	AT	TTCTGACCGTGTCCGATTGT	ATCACTCCGAGTTGGCTTGG	57	187
BPssr_21	TAT	TGAGATTGGCCCCTTCGAAC	CCGTATCCAGAAGAACGCGA	56	177
BPssr_22	CCT	ACGTTCTCACCGCTTCACTT	TCCGCCACTTCGATTTTCCA	57	193
BPssr_23	CCG	TCTCGTGAAACATGGACGGG	GCTAATGGGCTGCGGTTTTT	58	173
BPssr_24	GGC	ACTTTGGCTCGATCGAAGGG	ATCCCAGGAAGCCATTGACG	58	162
BPssr_25	ATT	TCAATTGACGTGGGCACTGT	GATCAGACCAGCCCACCTTC	58	241
BPssr_26	ATT	CGACGTGTCGCGCAATTTAT	ACCCAACCTGCACTCGAATT	54	208
BPssr_27	TAT	TAAACAGCAAGGCCCCAAGT	ACCAAAAATTCCACGGCAGC	55	234
BPssr_28	ATT	CATCCATAATGTCCCCGGCA	GGAGCGACCAGTAGTGATGG	55	272
BPssr_29	ATT	TGCATGCGTACCTTCACCTT	AAGTGCATCACAATGGCCCT	57	245
BPssr_30	ATT	TCCTTGTTTGGAGGGGAGGA	GGATGCAAATCAATGGCCGG	57	244
BPssr_31	TTA	GCGCTGCTGACATCAATGAG	TACAGCGTAGGTTTGCACGT	57	163
BPssr_32	ATT	AAGCTTGATGCCTTCCCTCA	TGACATCCAAATCTGGCCGT	57	250
BPssr_33	TTA	GAGTTCCACCACCAGCTACC	ATTACATAAGCCGGCTCGCA	57	226
BPssr_34	TAA	GTGACAAGAAGCTTCGCTGC	TCAGCCTTCAAGAGAGGGGA	57	207
BPssr_35	TTA	AAAAGGGTATGGGATGGCGG	TAGGCACGTAGAAGCAGTGC	55	160
BPssr_36	TAT	GGTTGGGGCACAAGTAGGTT	TGGATCGGGAGGTGTGGTAT	55	157
BPssr_37	AGA	GCACATGAAGCCATTCGACC	ATCCAGTACACCAGCCAACG	57	174
BPssr_38	TTA	ACGCACAAAGCATGCATGAG	TGCGCACAGATTAGCCTTCA	57	275
BPssr_39	AAT	CCTACAGAGGTTGCAGCACT	ATGGGTGCCGGTCCTCTATA	56	220
BPssr_40	TAA	TCTGCTCTTGATGGTGGCAG	ACACGTGTCAGGAAATCCCC	58	262
BPssr_41	(TA)T(TA)	TTGAACCCACAACCTTGAGG	GTGGTGACAGATAGGGCTGC	55	207
BPssr_43	(ATAG)(AT)A(AT)	TTCTCATTCACACGTGCACG	CTTGCAAGTCATGGCATGCA	55	186
BPssr_44	AATA	ACTCTGGAGCCTACATCGGA	TGCACATGTCTCGTTTTGCA	57	156
BPssr_45	TA	TCAACAGGATGAGCTAATGGGA	GACTGAATCGTTGGCCTGGA	55	201
BPssr_48	TTA	TGGTTTGTGGTAGATTCAGACT	ATGAGTCGAGGCAAATGCTG	57	227
BPssr_49	(AT)A(AT)	AAATGCAATTAGGGGCCACC	CTAGACGGAGGAGCAACGAG	55	183
BPssr_50	(TC)(TA)	TGGACGGCCTAGATTTGCTG	AGGTCGTTGCAACATTTAGTGT	57	196
BPssr_51	AT	CCCCAACAAACCATTTGGCA	TTTTGAGAGGAGCCAAGCCG	55	195
BPssr_54	AT	GGACGTTGGCTAGGCTCTTA	GATGCTAATGGAGACGCCGT	57	211
BPssr_55	(TTC)(TCT)	TTTTAACTCGACCGTGCCGA	ATGCTGTCCTGAGGTTGGTG	57	199
BPssr_56	(TA)(TTTA)	TCAGCTCTTTTTCAACCGCT	AAACAAGGATTCCACTCATAGATATTT	57	285
BPssr_57	TATT	ACTCTCCCTCTTTGCTTGGC	TCAGTTCAAAAACCAACAAGGGA	55	170
BPssr_58	(TATC)(TA)	GCTGACTTTGTGCCCAACAT	CAAGATAAGCTGGAGGGGGC	57	251
BPssr_60	AC	CACACACACCCTCCCATGAA	TTTCCTCAGGGAGCTGTTGA	57	196
BPssr_61	(TC)(AC)	CACACACACACACACACAGG	TTTGGATACGCGGGGTAAGC	57	196
BPssr_62	AT	GCGGGTAAGGATTTCTGCCT	TGTGTGTGTGAGGGCCATTT	57	201
BPssr_64	TA	GGCAGTGTTCGACTCGGTTA	AATGGGCTCGAGATGGAACG	57	227
BPssr_65	AAT	CACACCATGCAGACAACTGT	TCGTCGGTACAAGATGAACCA	53	290
BPssr_66	TAA	CACACCATGCAGACAACTGT	TCGTCGGTACAAGATGAACCA	53	245
BPssr_67	TA	AAGCCAATCGCATTAAGCCA	AAAGCCAGAACCTAGGTGCC	57	202
BPssr_70	GCG	AACTGTTGAAACTGCTGCCG	ATAAGTAAATTCGCCGCCGC	53	206
BPssr_71	AAT	AGGCCTCAAAAAGTGCAGGA	ATCAATCTTGCTCGGGGCTT	53	239
BPssr_72	TTA	ACGTCATCAATCCGAGCTTGT	GAGCCAAGCCAACCCAAAAT	53	204
BPssr_73	ATCACG	CGATCACGATCACGATCACG	ACGAACAGAGTCGAGGAGGA	57	199
BPssr_75	TTA	CCCACCGAGTCGAACGTTAT	TCTGATGAGACACCCACAACT	57	230
BPssr_77	TA	TATTGCCTCCCAAGAAGCGC	ACAGTTTTCCCACATGGTGC	57	191
BPssr_78	ATA	TCTTGCACCTTTCTGATTGCA	ACAGCTTGCTCTTAATGTTACTCT	57	200
BPssr_79	(AT)(AGTT)	GCCATGTAGAGCGATCTGGT	TCTTGCTCATGTTAGCTCACGT	57	233
BPssr_80	(TG)(TA)	CCAACCTGTCCACACAAGGA	TCCGGACCAGTAACACTTGT	57	169
BPssr_81	AT	AGTAGTGAGCGAATGAGGCT	CTGGCGCACGTCAACTTTAT	53	223
BPssr_82	TAA	TGGGTTAAGTGCTGGTAGTGT	ACTTGCATTATTGACATGAACATCA	53	205
BPssr_83	TAT	CTTCGACTTCCCCTGGTCTG	GTCGGTGCTACAACTGTGGA	57	152
BPssr_84	CCT	TCCAAGAAGCGCATCATCCA	GGTTCGACACTTGGTCCGTT	57	197
BPssr_85	TA	CCAACGGGAATGGAACAACC	GGAGCTCGTCACCTATGTGG	57	198

**Fig 7:**
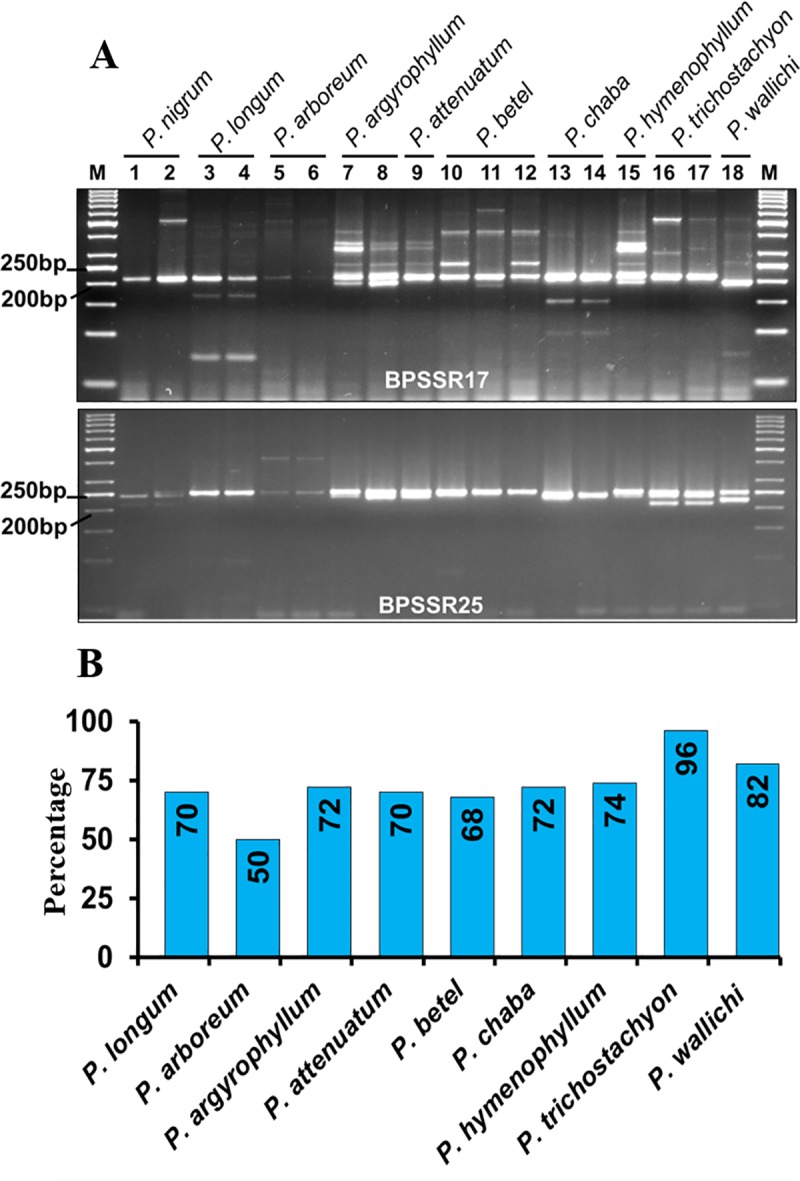
Cross-species transferability of SSR from *P*. *nigrum* in other species of *Piper*. **A**. Amplification profile of SSRs in different *Piper* species with primer BPSSR17 and BPSSR25. Name of the species have been mentioned in the top of the gel image. The numbers under the species name indicate different accessions from the species. M is the DNA molecular weight standard, 50bp ladder (MBI Fermentas). Primer names have been indicated at the bottom of each gel. **B**. Species wise transferability of SSRs from *P*. *nigrum* in nine different *Piper* species.

Further, cross species transferability of 50 SSR loci was checked in nine other *Piper* species namely, *P*. *longum*, *P*. *arboreum*, *P*. *argyrophyllum*, *P*. *attenuatum*, *P*. *betel*, *P*. *chaba*, *P*. *hymenophyllum*, *P*. *trichostachyon and P*. *wallichi*. One to three accessions from each *Piper* species were included in the study. The amplification profile of 18 black pepper accessions, representing nine *Piper* species and *P*. *nigrum* using two representative SSR primers have been shown in Fig 7A. Out of 50 SSRs screened, 19 primer pairs produced amplification in all nine other *Piper* species (Table 4). Total 31 primers showed amplification in at least seven *Piper* species. Among nine *Piper* species, highest rate of SSR transferability of *P*. *nigrum* was observed in *P*. *trichostachyon (96%)* followed by *P*. *wallichi* (82%); whereas least transferability was seen in *P*. *arboreum* (50%) (Fig 7B). Out of 50 SSRs tested for cross species amplification, 39 primers were polymorphic in nature with respect to the allele size amplified in *P*.*nigrum*.

**Table 4:** Cross species amplification of 50 SSRs from *P*. *nigrum* in 9 other *Piper* species. Numbers in the first row indicate species name and TCR number as given below. 1: *P*. *nigrum (*TCR419), 2: *P*. *nigrum* (TCR8), 3: *P*. *longum* (P25), 4: *P*. *longum* (TCR267), 5: *P*. *arboreum* (TCR267), 6: *P*. *arboreum* (*P*. *arboreum*), 7: *P*. *argyrophyllum* (TCR302), 8: *P*. *argyrophyllum* (TCR365), 9: *P attenuatum* (TCR171), 10: *P betel* (TCR166), 11: *P betel* (TCR357), 12: *P betel* (Lakshdweep), 13: *P chaba* (TCR149), 14: *P*. *chaba* (TCR265), 15: *P*. *hymenophyllum* (TCR345), 16: *P*. *trichostachyon* (TCR363), 17: *P*. *trichostachyon* (CR279), 18: *P*. *wallichi* (*P*. *wallichi*). Plus (+) and minus (-) sign indicate cross species transferability. Monomorhic/polymorphic indicate amplification of same/different allele as *P*. *nigrum*.

S. No.	SSR marker	1	2	3	4	5	6	7	8	9	10	11	12	13	14	15	16	17	18	Nature
1	**BPSSR1**	+	+	+	+	-	-	-	-	+	+	+	-	-	-	-	+	+	+	Polymorphic
2	**BPSSR2**	+	+	-	-	-	-	-	-	-	-	-	-	-	-	-	-	-	-	Monomorphic
3	**BPSSR3**	+	+	+	+	+	+	+	+	+	+	+	+	+	+	+	+	+	+	Polymorphic
4	**BPSSR4**	+	+	+	+	-	-	+	+	+	+	+	+	+	+	+	+	+	+	Polymorphic
5	**BPSSR5**	+	+	-	-	-	-	-	-	-	+	+	+	+	+	-	+	+	+	Polymorphic
6	**BPSSR6**	+	+	-	-	-	-	-	-	-	-	-	-	+	+	+	+	+	-	Polymorphic
7	**BPSSR7**	+	+	-	-	+	+	-	-	-	-	-	+	-	-	-	+	+	+	Polymorphic
8	**BPSSR9**	+	+	+	+	-	-	-	-	-	+	+	+	+	+	-	+	+	+	Polymorphic
9	**BPSSR10**	+	+	+	+	-	-	+	+	+	+	+	+	-	-	+	+	+	+	Polymorphic
10	**BPSSR11**	+	+	+	+	+	+	+	+	+	+	+	+	+	+	+	+	+	+	Polymorphic
11	**BPSSR12**	+	+	+	+	-	-	+	+	+	-	-	-	+	+	+	+	+	+	Monomorphic
12	**BPSSR13**	+	+	+	+	-	-	+	+	+	-	-	-	+	+	+	+	+	-	Polymorphic
13	**BPSSR14**	+	+	-	-	-	-	-	-	-	-	-	-	-	-	-	+	+	+	Monomorphic
14	**BPSSR15**	+	+	-	-	-	-	+	+	+	+	+	+	+	+	-	+	+	+	Polymorphic
15	**BPSSR16**	+	+	-	-	+	+	+	+	+	+	+	+	+	+	+	+	+	+	Polymorphic
16	**BPSSR17**	+	+	+	+	-	-	+	+	+	+	+	+	+	+	+	+	+	+	Polymorphic
17	**BPSSR18**	+	+	-	-	-	-	+	+	-	-	-	-	-	-	+	+	+	+	Polymorphic
18	**BPSSR19**	+	+	-	-	-	-	+	+	+	-	+	-	+	+	+	+	+	+	Polymorphic
19	**BPSSR20**	+	+	+	+	-	-	+	+	-	+	+	+	+	+	+	+	+	-	Polymorphic
20	**BPSSR21**	+	+	+	+	+	+	+	+	+	+	+	+	+	+	+	+	+	+	Polymorphic
21	**BPSSR22**	+	+	+	+	+	+	+	+	+	+	+	+	+	+	+	+	+	+	Polymorphic
22	**BPSSR23**	+	+	+	+	-	-	-	-	-	-	-	-	+	+	-	+	+	+	Polymorphic
23	**BPSSR24**	+	+	+	+	+	+	+	+	+	+	+	+	+	+	+	+	+	+	Polymorphic
24	**BPSSR25**	+	+	+	+	+	+	+	+	+	+	+	+	+	+	+	+	+	+	Polymorphic
25	**BPSSR26**	+	+	-	-	-	-	+	+	-	-	-	-	-	-	+	+	+	+	Polymorphic
26	**BPSSR27**	+	+	+	+	+	+	+	+	+	+	+	+	+	+	+	+	+	+	Polymorphic
27	**BPSSR29**	+	+	+	+	+	+	+	+	+	+	+	+	+	+	+	+	+	+	Polymorphic
28	**BPSSR30**	+	+	+	+	+	+	+	+	+	+	+	+	+	+	+	+	+	+	Monomorphic
29	**BPSSR31**	+	+	+	+	-	-	-	-	-	-	-	-	-	-	-	-	-	+	Monomorphic
30	**BPSSR32**	+	+	+	+	+	+	+	+	+	+	+	+	+	+	+	+	+	+	Polymorphic
31	**BPSSR33**	+	+	+	+	+	+	+	+	+	+	+	+	+	+	+	+	+	+	Polymorphic
32	**BPSSR36**	+	+	+	+	+	+	+	+	+	+	+	+	+	+	+	+	+	+	Polymorphic
33	**BPSSR37**	+	+	+	+	+	+	+	+	+	+	+	+	+	+	+	+	+	+	Monomorphic
34	**BPSSR38**	+	+	+	+	+	+	+	+	+	+	+	+	+	+	+	+	+	+	Polymorphic
35	**BPSSR40**	+	+	-	-	+	+	+	+	+	+	+	-	+	+	+	+	+	-	Polymorphic
36	**BPSSR44**	+	+	-	-	-	-	+	+	+	+	-	-	-	+	+	+	+	+	Polymorphic
37	**BPSSR50**	+	+	+	+	+	+	+	+	+	+	+	+	+	+	+	+	+	+	Polymorphic
38	**BPSSR55**	+	+	+	+	-	-	+	+	+	-	-	-	-	-	+	+	+	-	Polymorphic
39	**BPSSR57**	+	+	+	+	+	+	+	+	+	+	+	+	-	-	+	+	+	+	Monomorphic
40	**BPSSR61**	+	+	-	-	-	-	-	-	-	-	-	-	-	-	-	+	+	-	Monomorphic
41	**BPSSR62**	+	+	-	-	-	-	-	-	-	-	-	-	-	-	-	+	+	-	Monomorphic
42	**BPSSR67**	+	+	+	+	+	+	+	+	+	+	+	+	+	+	+	+	+	+	Polymorphic
43	**BPSSR71**	+	+	+	-	-	-	+	+	+	+	+	+	+	+	+	+	+	+	Polymorphic
44	**BPSSR73**	+	+	-	-	+	+	-	-	-	-	-	-	-	-	-	+	+	+	Polymorphic
45	**BPSSR75**	+	+	+	+	+	+	+	+	+	+	+	+	+	+	+	+	+	+	Polymorphic
46	**BPSSR77**	+	+	+	+	+	+	+	+	+	+	+	+	+	+	+	+	+	+	Monomorphic
47	**BPSSR78**	+	+	+	+	-	-	-	-	-	-	-	-	-	-	-	+	+	-	Monomorphic
48	**BPSSR80**	+	+	+	+	-	-	+	+	+	+	+	+	+	+	+	+	+	+	Polymorphic
49	**BPSSR83**	+	+	+	+	+	+	+	+	+	+	+	+	+	+	+	+	+	+	Polymorphic
50	**BPSSR85**	+	+	+	+	+	+	-	-	+	+	+	+	+	+	+	+	+	+	Polymorphic

## Discussion

SSR markers have played pivotal role in genetic analysis, mapping, gene tagging and marker assisted breeding in several crop plants. Availability of SSRs in crop plants have in a way proved a ‘stepping stone’ for the rapid genetic dissection of complex traits including resistance to biotic and abiotic stress, identification of QTLs for several important traits, genetic enhancement and varietal development. In spite of immense economic importance, black pepper lacked abundance of SSRs in general and genic SSRs in particular. The present reports of genomic SSRs in black pepper would meet researchers/geneticist/plant breeders requirement and therefore is expected to pave way forward for downstream application in genetic dissection, diversity studies, QTL identification, marker assisted breeding etc.

In recent past, SSRs have been reported in black pepper using transcriptome approach [23–24]. SSRs identified using transcriptome based approach gives information only about expressed region of the genome leaving large inter genic region unrepresented thus limiting pan genome applications. Such EST-SSRs although have been useful for genetic analysis in other plants species, however, were found to be relatively low polymorphic and concentrated in gene-rich regions of the genomes, which could limit their applications especially in the linkage maps construction [45–48]. On the other hand, the genomic SSRs are highly polymorphic and have pan genome distribution which facilitates the better map coverage [45, 48–49]. Owing to their polymorphic nature over genic SSRs, intergenic SSRs were also found to have greater application in DNA fingerprinting and varietal identification [50].

Among the different SSRs identified, frequency of the dinucleotide was highest in the *P*. *nigrum* genome. Predominance dinucleotide SSRs among the other SSRs was also observed in other plants such as Mung bean, Cranberry, Pigeonpea, black alder and Maqui [32, 51–53]. The next abundant SSR were with tri, tetra and hexa repeats and the least abundant SSRs were with penta nucleotide repeats. Frequency of identified SSR in the Black pepper genome is 158/MB (one SSR for every 6.3 kb region of DNA) is significantly high compared to other plants genomes such as of *Gossypium* species. In the assembled genome of *G*. *hirsutum*, *G*. *arboreum*, and *G*. *raimondii* frequency of SSR is reported as one every 24.3, 20.4 and 13.4 Kb, respectively [28].

Microsatellites are markers of choice for diversity analysis, mapping and other genetic studies because of their abundance, reproducibility and polymorphism. In black pepper, there are relatively less number of diversity studies reported using SSRs compared to other crop plants. In the present study, from the identified SSRs, 50 SSRs were used to study diversity in 30 germplasm accessions of black pepper, mostly from the Kerala state of India. The primers were validated in a set of 30 *P*. *nigrum* accessions. Total number of alleles detected at 50 loci was 215 with an average number of 4.3 alleles per locus. The dendrogram generated using SSR data grouped 30 landraces into four clusters. The clustering pattern does not unravel any relationship between genetic similarity and the place of origin/collection. Overall these markers detected high level of polymorphism and high diversity among accessions of *P*. *nigrum* studied. Cross transferability of these markers was checked across nine species and 39 out of 50 primers were polymorphic with respect to the allele size amplified in *P*. *nigrum*. In the recent past, few microsatellite markers have been developed and used for different studies in black pepper. Earlier nine microsatellite markers were developed and characterized from an enriched library of *P*. *nigrum* and tested for transferability in four distinct *Piper* species [22]. Out of nine SSRs, five produced amplification in all four species tested. In another study, the molecular characterization carried out using SSR markers could demarcate Indian and exotic *Piper* species [54]. Genetic diversity assessed using 13 EST SSR markers detected high genetic diversity among 148 black pepper germplasm [13]. These reports are in congruence with the present study in black pepper where a high genetic diversity is observed among the 30 landraces of black pepper at 50 identified SSR loci.

The genomic microsatellite markers identified in black pepper in this study would form valuable and long awaited resources for researchers/plant breeders for its wide applications in diversity studies, linkage mapping, evolutionally biology, DNA fingerprinting, trait association study etc. in near future, paving the way for harnessing the potential of marker assisted breeding in black pepper genetic enhancement and improvement.

## Conclusion

Non-availability of sufficient number of polymorphic SSRs in black pepper necessitated identification of new markers and their characterization. The recently sequenced genome of black pepper by our group was used for identification and validation of SSRs. Total 69,126 SSRs with frequency of 158 per MB were mined from the assembled draft genome, would fulfil the deficiency of genomic SSRs in black pepper. Validation of the identified SSRs on a set of 30 accessions of *P*. *nigrum* and their cross species transferability to nine species shows the potential application of the identified SSR markers not only in *P*. *nigrum* but also in other species of *Piper* where genomic resource is still scarce.

## Supporting information

S1 Table List and sequences of primers of black pepper SSR. (XLSX)
